# A multi-level qualitative analysis of Telehomecare in Ontario: challenges and opportunities

**DOI:** 10.1186/s12913-015-1196-2

**Published:** 2015-12-09

**Authors:** Gemma Hunting, Nida Shahid, Yeva Sahakyan, Iris Fan, Crystal R. Moneypenny, Aleksandra Stanimirovic, Taylor North, Yelena Petrosyan, Murray D. Krahn, Valeria E. Rac

**Affiliations:** Toronto Health Economics and Technology Assessment (THETA) Collaborative, University of Toronto, Leslie Dan Pharmacy Building, 144 College Street, Toronto, M5S 3M2 ON Canada; University of Toronto, Toronto, ON Canada; Leslie Dan Faculty of Pharmacy, University of Toronto, 144 College Street, Toronto, M5S 3M2 ON Canada; Bloomberg Faculty of Nursing, University of Toronto, 155 College Street, Toronto, M5T 1P8 ON Canada

**Keywords:** Telehomecare, Telehealth, qualitative research, implementation, adoption, barriers, multi-level, heart failure, chronic obstructive pulmonary disease, chronic disease management

## Abstract

**Background:**

Despite research demonstrating the potential effectiveness of Telehomecare for people with Chronic Obstructive Pulmonary Disease and Heart Failure, broad-scale comprehensive evaluations are lacking. This article discusses the qualitative component of a mixed-method program evaluation of Telehomecare in Ontario, Canada. The objective of the qualitative component was to explore the multi-level factors and processes which facilitate or impede the implementation and adoption of the program across three regions where it was first implemented.

**Methods:**

The study employs a multi-level framework as a conceptual guide to explore the facilitators and barriers to Telehomecare implementation and adoption across five levels: technology, patients, providers, organizations, and structures. In-depth semi-structured interviews and ethnographic observations with program stakeholders, as well as a Telehomecare document review were used to elicit key themes. Study participants (*n* = 89) included patients and/or informal caregivers (*n* = 39), health care providers (*n* = 23), technicians (*n* = 2), administrators (*n* = 12), and decision makers (*n* = 13) across three different Local Health Integration Networks in Ontario.

**Results:**

Key facilitators to Telehomecare implementation and adoption at each level of the multi-level framework included: user-friendliness of Telehomecare technology, patient motivation to participate in the program, support for Telehomecare providers, the integration of Telehomecare into broader health service provision, and comprehensive program evaluation. Key barriers included: access-related issues to using the technology, patient language (if not English or French), Telehomecare provider time limitations, gaps in health care provision for patients, and structural barriers to patient participation related to geography and social location.

**Conclusions:**

Though Telehomecare has the potential to positively impact patient lives and strengthen models of health care provision, a number of key challenges remain. As such, further implementation and expansion of Telehomecare must involve continuous assessments of what is working and not working with all stakeholders. Increased dialogue, evaluation, and knowledge translation within and across regions to understand the contextual factors influencing Telehomecare implementation and adoption is required. This can inform decision-making that better reflects and addresses the needs of all program stakeholders.

**Electronic supplementary material:**

The online version of this article (doi:10.1186/s12913-015-1196-2) contains supplementary material, which is available to authorized users.

## Background

Chronic obstructive pulmonary disease (COPD) and heart failure (HF) impose a significant burden on individuals and health care systems worldwide [1, 2]. COPD is the leading cause of death in Canada and HF has been estimated to affect half a million Canadians [3, 4]. These chronic diseases contribute to a high rate of avoidable hospitalizations and in turn, health system costs [5, 6]. Promisingly, telehealth interventions delivered in the home or Telehomecare, have shown potential in reducing hospitalizations [7–9] and improving quality of life, self-management and access to care for people with COPD, HF and other chronic diseases [10–13].

Evidence demonstrating the effectiveness and cost-effectiveness of Telehomecare however, remains mixed and difficult to synthesize. This is likely due to the contextual heterogeneity of studies evaluating Telehomecare, across factors including the type of intervention, the participants involved, and the health care system in which they are located [14, 15]. Importantly, qualitative research has begun to interrogate the role of context – from the individual level to the organizational level – and how this can shape the perception, implementation and effectiveness of Telehomecare [16–21].

This article discusses the qualitative component of a three-part mixed-method program evaluation of Telehomecare in Ontario, Canada, commissioned by the Ontario Ministry of Health and Long-Term Care (MOHLTC). The objective of the qualitative component was to comparatively explore the multi-level factors and processes which facilitate or impede the implementation and adoption of the program across three Local Health Integration Networks (LHINs). This evaluation is timely as local-level data has shown the potential for this program to reduce hospital admissions and emergency department visits [22], yet a comprehensive evaluation of its implementation, adoption, effectiveness and cost effectiveness has not been conducted.

This qualitative study builds on and addresses gaps in the evidence base on Telehomecare. It employs a unique multi-level analysis to explore the facilitators and barriers to Telehomecare implementation and adoption across five levels spanning from micro to macro: technology, patients, providers, organizations, and structures. It is also the first known study to include the perspectives of a wide range of Telehomecare stakeholders, including patients, informal caregivers, health care providers, technicians, administrators, and decision makers across different health regions. Such qualitative examination is vital to inform the current Telehomecare evidence base which is largely focused on outcomes rather than on the spectrum of people and processes that can shape Telehomecare [17, 20, 23, 24]. Understanding and addressing these factors is particularly pressing, given that implementation has often been challenging, in Canada and internationally [18, 25–28].

### The Telehomecare program in Ontario

In 2007, the Ontario Telemedicine Network (OTN) launched the largest Telehomecare program piloted in Canada. By working with eight Family Health Teams, 800 patients with COPD and HF were enrolled in the pilot program. Following this, the ‘first wave’ of program expansion began in 2012 across three LHINs: North East (NE), Toronto Central (TC), and Central West (CW). Since then (and as of July 2015), 6,334 patients in these LHINs have been referred to the program and a second wave of expansion across additional LHINs is now underway. Each LHIN oversees Telehomecare program planning and implementation and contains a host organization (e.g., a Community Care Access Centre [CCAC] or hospital) which facilitates access to the program at the community level.

The Telehomecare program has two goals: to increase self-management skills for patients with COPD and HF; and to improve the monitoring of these patients via remote health status monitoring. The program has a six month duration and involves: i) designated Telehomecare nurses (within the host organization) with whom patients and/or informal caregivers can interact by telephone; ii) daily transmission of patient data (weight, blood pressure, oxygen levels, and answers to daily questionnaire) to a Telehomecare nurse via a remote monitoring device; iii) individualized care based on patient needs (e.g., following up with patient if their data is outside of a normal range and weekly coaching sessions); and iv) communication regarding patient health concerns between the Telehomecare nurse and other members of the patient’s circle of care.

## Methods

### Conceptual framework

**Figure 1:**
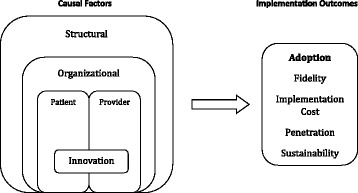
A multi-level framework predicting implementation outcomes. Modified from Chaudoir et al. Implementation Science 2013, 8:22

Chaudoir and colleagues’ [29] *multi-level framework for implementation research* (Fig. 1) was employed as a conceptual guide to capture the factors that influence the implementation and adoption of Telehomecare. This framework was developed to reflect a growing recognition that the effectiveness or cost-effectiveness of an innovation has only a partial influence on its uptake [29]. Rather, implementation is shaped by factors and processes across five levels: innovation-level (for our purposes, technology), patient-level, provider-level, organizational-level, and structural-level.

Aligning with the multi-level framework, the innovation-level includes factors or processes related to the innovation itself, specifically the Telehomecare technology used to monitor and communicate patient health information. Next, the patient-level includes characteristics and experiences of Telehomecare patients (e.g., motivation, perception of program, physical and/or mental abilities, etc.), while the provider-level similarly includes characteristics and experiences of health care providers involved in the provision of Telehomecare (e.g., beliefs, health care roles and capacities, etc.). Further, the organizational-level encompasses the factors and processes that relate to the organizations at which Telehomecare is being implemented (e.g., work climate or culture, staff dynamics, organizational protocol and practices, etc.). Lastly, the structural level encompasses societal factors and processes beyond the organizational level (e.g., sociocultural contexts, geography, public policies, etc.) [29].

As reflected in the framework (Fig. 1), the adoption of Telehomecare was the ‘implementation outcome’ of focus for this study because adoption occurs early in implementation processes as opposed to outcomes occurring later (i.e., sustainability) [29]. Adoption is considered to be the “intention, initial decision, or action to try or employ an innovation or evidence-based practice” and can also be referred to as “uptake” [30]. Adoption is an appropriate outcome for this evaluation of Telehomecare, given the program is still in its early stages in each LHIN (NE and TC LHINs began to enroll patients in mid-2012, and CW in early 2013) and there is an absence of data to date on other Telehomecare implementation outcomes.

### Study design

To explore the multi-level factors shaping the implementation and adoption of Telehomecare, the study employed: i) in-depth semi-structured interviews; ii) ethnographic observations; and iii) a review of documentary sources. This combination of data collection techniques was used to provide multiple sources of evidence for capturing the social complexity of Telehomecare.

### Study population & recruitment

The study sample included 39 patients and/or informal caregivers, 23 health care providers (i.e., 16 Telehomecare nurses and 7 primary care providers), two technicians, 12 administrators, and 13 decision makers across the three LHINs under study: NE, TC, and CW. An inclusion criterion for all study participants is outlined in Table 1 below.

**Table 1:** Study participant inclusion criteria

Stakeholder group	Inclusion criteria
Patients	• Consented to: a) participate in the program; and b) be contacted by the study team for evaluation purposes
	Telehomecare program patient eligibility criteria:
	• Diagnosed with HF or COPD (with or without co-morbid conditions) • A ‘heavy user’ of the health care system, characterized by any of the following: ○ A minimum of *one* hospitalization for a respiratory or cardiac complaint in the past six months ○ A minimum of *two* emergency department/urgent care center visits for a respiratory or cardiac complaint in the past six months ○ Receiving nursing services via CCAC ○ Frequent visits to primary care provider in the past year • Lives in a residential (private home or retirement home) setting with an active landline • Fluent in English (or their informal caregiver) • Able and willing to operate the Telehomecare equipment (or their informal caregiver) • Over 18 yrs of age (or their informal caregiver), and willing to provide informed consent.
Health Care Providers	• Referred a patient to the Telehomecare program (any health care provider) • Primary care provider of patient(s) enrolled in the study • Telehomecare nurses/physicians involved in the provision of care to patients enrolled in the Telehomecare program (must have 3 months experience with Telehomecare for interviews) • Over 18 yrs of age and able and willing to provide informed consent.
Technicians	• Involved in the set-up of Telehomecare equipment at patients’ homes • Over 18 yrs of age and able and willing to provide informed consent.
Administrators	• Administrators of the Telehomecare program as a larger network of care such as clinical service managers, program coordinators, etc. • Over 18 yrs of age and able and willing to provide informed consent.
Decision Makers	• Decision Makers involved in the Telehomecare program as a larger network of care such as regional program managers, key members of the LHIN, OTN etc. • Over 18 yrs of age and able and willing to provide informed consent.

The criteria for patient inclusion in the study was the same as the criteria for patient eligibility for the Telehomecare program. The potential patient population had consented to be contacted for evaluation purposes at the time of enrollment into the Telehomecare program (*n* = 2,916) between June 28^th^ 2012 and December 31^st^ 2014. Only 1.5 % of the total patient population enrolled during this time chose not to be contacted for evaluative purposes. The contact information of potential patients (including current, former, and patients who had left the program before completion) was accessed using the Patient Monitoring and Management System (PMMS) managed by the OTN. Patient information was extracted from PMMS and entered into a participant screening log for the purpose of contacting potential patients. The recruitment of patient participants from each LHIN (15 from NE, 10 from CW, and 14 from TC) was based on purposeful sampling. This means that patient selection was based on an iterative process that sought to maximize the richness of the research data until thematic saturation was reached (no new data was emerging) [31]. In particular, the study team sought the inclusion of patients from varied locations within each LHIN, to gain insight into how Telehomecare compares and contrasts across health systems and geographies. Details of patient participants are outlined in Table 2 below.

**Table 2:** Patient participant information

PT ID	Gender	Age	Diagnosis	LHIN	PT ID	Gender	Age	Diagnosis	LHIN
PT 018	Female	61	COPD	CW	PT 110^+^	Male	92	HF	TC
PT 030	Male	83	COPD	CW	PT 125	Male	75	NA	NE
PT 031	Male	80	HF	CW	PT 128	Female	65	COPD	CW
PT 032	Male	71	HF	CW	PT 133	Male	79	HF	NE
PT 039^+^	Female	77	COPD	TC	PT 134	Male	65	NA	NE
PT 044	Female	76	COPD	NE	PT 135^+^	Female	79	HF	NE
PT 052	Female	84	COPD	TC	PT 136^+^	Female	67	COPD	NE
PT 053	Male	78	COPD	TC	PT 137^+^	Male	75	COPD	NE
PT 055	Female	50	COPD	TC	PT 138^+^	Female	80	COPD	NE
PT 073	Male	61	NA	NE	PT 139^+^	Female	78	HF	NE
PT 094^+^	Female	87	HF	CW	PT 140	Female	70	COPD	NE
PT 095^+^	Male	83	HF	CW	PT 141^+^	Male	51	HF	TC
PT 097^+^	Male	67	COPD	TC	PT 142^+^	Female	56	HF	TC
PT 098^+^	Female	37	HF	CW	PT 143^+^	Female	86	HF	TC
PT 099	Male	56	COPD	TC	PT 144^+^	Female	84	HF	TC
PT 102^+^	Male	69	COPD	TC	PT 148^a^	Male	76	COPD	CW
PT 103	Male	62	COPD	NE	PT 149	Male	75	COPD	TC
PT 106^+^	Female	71	HF	NE	PT 150^a^	Female	77	HF	NE
PT 107^+^	Male	73	COPD	CW	PT 152^+^	Male	94	HF	TC
PT 109^+^	Female	82	HF	NE					

^a^dropped out of program

^+^completed program

NA information not available

All other study participants (providers, administrators, decision makers and technicians) were introduced and referred to the study team over email or through introductory meetings arranged with the assistance of designated Telehomecare Engagement Leads from each LHIN. The OTN played an integral role in liaising and facilitating communication during these early stages of recruitment. Introductory meetings were held at the beginning of the study in each LHIN, in person and via teleconference, and allowed the study team to explain the program evaluation to stakeholders. The study team was referred to Telehomecare Nurses by the LHIN Engagement Leads and physicians who had referred patients to the program and/or had patients who were enrolled in the program were also invited to participate. A combination of purposeful and snowball sampling was used to ensure maximal variation in sampling across the LHINs of focus. Similar to patient recruitment, stakeholders across various regions and decision-making levels were sought. Details on the sample sizes of participant groups from each LHIN are provided in Table 3 below.

**Table 3:** Conducted observations & interviews

Method/ Stakeholder	Central West	Toronto Central	North East
Observations (total)	~10 h	~15 h	~8 h
Interviews (total)	25	26	34
HCPs (total)	7	8	8
Physicians	2	3	2
Nurses	5	5	6
Administrators	5	2	5
Decision Makers	3	2	6
Patients	10	14	15
Technicians	2 (non-specific LHIN)		
Decision Makers	2 (non-specific LHIN)		

### Data collection

Data collection for this study took place between June and December 2014. Data triangulation occurred in two ways. First, methodological triangulation (or employing multiple data collection techniques) occurred using semi-structured interviews, ethnographic observations, and a review of Telehomecare related documents (see Table 3 below). Second, data source triangulation occurred by seeking information from multiple stakeholder groups. The collection of data using multiple techniques from different sources has increasingly been recognized as strengthening the credibility and the transferability of health research findings [17, 32]. Specifically, this allowed for converging and diverging lines of inquiry to develop during the process of data collection.

#### Semi-structured interviews

In-depth semi-structured interviews were conducted with 89 study participants, some being interviewed in pairs or a set of three (*n* = 9). Interviews were held in person where feasible (*n* = 36), within the home setting of patients and work setting of other stakeholders, and by telephone (*n* = 48). They ranged from 30 min to one hour, and were recorded and transcribed verbatim. Interview notes were taken at the request of six patients who preferred not to be recorded. Semi-structured interview guides aided in the exploration of stakeholder experiences with and perspectives on the program (see Additional file 1). These guides were developed as tools for ethnographic inquiry, with questions intended to be open-ended and non-leading allowing for interview participants to describe their thoughts and experiences freely and for new lines of inquiry to emerge from the dialogue that may be relevant to the research. Grounded theory - which posits that theories of certain phenomena emerge and shift with the process of data collection and analysis – informed interview guide development. Specifically, interview questions were designed to explore meaning and/or generate hypotheses rather than prove a priori theories of what is most relevant. Further, as potential key theories and themes arose in the preliminary data analysis, some interview questions were modified or added to generate more information on potential emerging themes [33, 34].

#### Ethnographic observations

Ethnographic observations took place alongside in-person interviews, with the exception of two observation sessions in which the stakeholders had already been interviewed by phone. Patients who were no longer in the program were not observed, as they could not demonstrate technology use. Approximately 33 h of observation occurred across 29 stakeholders, with all impressions documented through field notes. Ethnographic observations provided insight into the day-to-day lives and environments of Telehomecare stakeholders. They also allowed for in-person connection and informal dialogue to occur between the interviewer and the study participants, allowing for issues to be discussed more candidly than they might be in a semi-structured interview [35]. Importantly, observations revealed potential areas of convergence or divergence between what was said by participants and what was observed. In this, observations contributed to important insights that may not have been gleaned from interviews alone. Observation notes reflected the following dimensions:

- Living and working spaces (e.g., accessibility, safety, proximity to people and resources, etc.);
- Daily activities (e.g., ease of technology use for patients, roles and responsibilities, etc.); and
- Interactions (e.g., how technicians train patients and/or informal caregivers to use Telehomecare technology, how nurses interact with each other, their patients, other administrators, etc.)

#### Document review

Lastly, a document review was conducted, focusing on Telehomecare nursing materials, publicly available Telehomecare-related documents, and patient education literature. OTN provided the research team with Telehomecare certification materials (i.e., Telehomecare nurse reference and training binders for Telehomecare nurses and coordinators). They also provided access to two electronic resource libraries for Telehomecare providers that are linked from the central OTN Telehomecare website (http://Telehomecare.otn.ca/). These libraries contain current training and education material, program documents, and general reminders for Telehomecare patient care. Other Telehomecare documents (i.e., OTN presentations, reports, etc.) were found on the same website, which is the primary hub of information on the Telehomecare program for prospective and current patients, health care providers, and the general public. Throughout data collection, further materials shared by stakeholders (i.e., patient education literature; technician installation instructions) were also reviewed as well as new documents posted online.

### Data analysis

Qualitative data analysis took place alongside data collection. Analysis was an iterative process, and undertaken using the Grounded Theory method outlined by Glaser and Strauss [36] and Strauss and Corbin [37]. This involved systematic coding of data and theme abstraction with the goal of identifying key facilitators and barriers to Telehomecare implementation and adoption in relation to the multi-level framework. The multi-level framework facilitated a critical conceptualization of the program as influenced by multiple level factors and allowed for comparisons and contrasts within each level to be identified across regions. Classification of major facilitators and barriers to Telehomecare across multiple levels and regions importantly ensured that our findings could inform recommendations appropriate to their context.

Thematic analysis of the interview transcripts, interview notes, and observation notes occurred in three stages: open coding (data reduction), axial coding (data display) and selective coding (conclusion drawing) [38]. Open coding of the data was led by the qualitative lead, with members of the research team independently coding interview transcripts and notes, and observation notes into descriptive and overarching codes (e.g., use of technology, access to health care, etc.). Initial codes were shared with the lead qualitative researcher after this first phase, which allowed for comparison and refinement of codes into key themes. Variances in coding among the team allowed for alternative explanations of the data to be explored, ensuring substantive interpretation of the data [39].

Axial coding conducted by the qualitative lead involved close analysis and comparison of all descriptive codes, which allowed for new critical codes to emerge, including conflicting codes. All codes were then classified according to a particular level in the multi-level framework, with the recognition that some codes cut across more than one level. Interviews and observations occurred until thematic saturation of the data was reached (no new codes were emerging). This ongoing data collection allowed for emerging themes to be further explored in interviews with stakeholders. All final themes were informed by continuous dialogue among the research team. This dialogue facilitated self-reflection on how the analysis evolved which allowed for the lead analyst to fully interrogate potential assumptions or biases reflected in their interpretation of the data [40].

The Telehomecare documents reviewed provided important contextual insight in relation to study participant perspectives and experiences. In the preliminary phases of the research, these documents provided a basic knowledge about the program and the types of resources and materials available and used by Telehomecare stakeholders. Similar to our interview and observation analysis, documents were read and revisited to gain a full understanding of program resources, protocols, and contexts. Importantly, triangulated data analysis between interview transcripts and notes, observation notes, and Telehomecare documents allowed for an in-depth comparative exploration of convergence and dissonance in the data [41]. This allowed for potential disjunctures and gaps between levels to be seen (e.g. between institutional protocol and Telehomecare nurse experiences) and ensured robustness and reliability of research findings. Reliability of the research findings was also strengthened by maintaining a chain of evidence [42] throughout the study. This ensured that the evolution of the qualitative results could be followed by an external observer, to ensure credibility of the data collection and analytical process.

### Ethical approval

Prior to engaging potential study participants, the program evaluation protocol was approved by the Research Ethics Board (REB) of the University of Toronto (Ref: #30158, 04 June 2014). Ethics approval was also obtained from 19 research sites including hospitals and CCACs, respective to the LHINs under evaluation. Potential patient participants were contacted by the evaluation study team by telephone, and if they chose to participate in the evaluation, a verbal consent was obtained with use of a telephone consent script approved by the REB. All other stakeholders who agreed to take part in the study provided consent in person or by telephone. Participants who consented received a copy of the study information and consent form and were aware that the research findings would be published within reports, articles, and presentations. They were given the option of being directly quoted within the consent form, and were guaranteed confidentiality and an anonymous presentation of the findings.

## Results

Overall impressions of Telehomecare across all stakeholders were positive. The program was often described as an innovative way to potentially improve patient self-management, health care, and well-being while decreasing visits to a primary care provider. However, a number of challenges to Telehomecare implementation and adoption were found, as well as potential opportunities to address these challenges. Results are summarized according to the following overarching themes that correspond to each level of the multi-level framework, from the micro- to macro-level. Each theme plays a key role in shaping Telehomecare implementation and adoption.

- Alignment between technology and individual contexts
- Alignment between patient abilities and program involvement
- Alignment between health care provider roles and capacities to fulfill them
- Alignment between organization objectives and the current health system
- Alignment between structural contexts and program operation

These themes are discussed below, as well as key facilitators and barriers that help or hinder alignment. Though presented as level-specific themes, many are interrelated and occur across more than one level. All illustrative quotes are identified according to stakeholder position and study identification number.

### Technology-level: alignment between technology and individual contexts

The Telehomecare program involves daily health status monitoring using a blood pressure cuff, pulse oximeter, floor scale, and touch-screen tablet. An overarching theme regarding Telehomecare technology was that its use and potential benefit were dependent on the individual contexts and needs of patients. User-friendliness was a key facilitator in technology use, whereas access-related issues presented barriers to use.

For the most part, patients and informal caregivers found the equipment straightforward to use and a simple way to quickly access health information. As testament to this, many patients wanted to purchase their own monitoring equipment before the end of their participation in the program. Some highlighted that the technology was intimidating at first, but with a technical demonstration and practice, it was easily used. This finding contradicted a common assumption of other stakeholders interviewed, that elderly people would likely resist technology use or have difficulties with it due to intimidation.

It was also acknowledged across stakeholders that Telehomecare technology has become more user-friendly over time for patients and informal caregivers:

*“There is a lot of feedback from the patients, from staff, to be able to have a tablet that is much more user friendly. Even the lighting, the size of the font, all of those things were things that were advised.” – Administrator 017*

Many stakeholders stated this technology evolution as being a natural part of implementing an innovation that had not yet been tested for that purpose.

However, the findings show that a key barrier to technology use was related to patient limitations in accessing or operating equipment. For instance, informal caregivers, of whom a large number participated in interviews and observations alongside patients, often helped patients use the technology. Informal caregiver assistance included reminding patients who might be experiencing poor memory how and when to take their readings, or helping patients with complex conditions physically use the equipment (e.g., to stand up on scale, to put on cuff, to read and answer questions on screen, etc.). Some informal caregivers struggled with taking on this task by themselves:

*“…she had to stand on a scale, and we tried a whole bunch of different ways to get that reading… The first couple of times we were able to do it with the help of a secondary person. If I was on my own with her, there was no way to get that reading.” – Informal Caregiver of Patient 144*

In addition, technicians highlighted that they were sometimes not aware of a patient’s inability to stand on the scale (a requirement for enrollment) until they went to the patient’s home to install and demonstrate the equipment:

*“I set out the equipment…and I say ‘Can you bring your mom? You can do it and I will watch’… And he said ‘but my mom can’t stand on the scale’… To stand on the scale you need to stand and be stable for a moment or two to take a reading… His mom couldn’t do that.” – Technician 070*

In these instances, patients have been removed from the program, while others that might not require daily weight monitoring (e.g., many COPD patients) have been told they do not have to monitor their weight. Overall, many stakeholders recommended that the technology be adapted to improve accessibility for people with disabilities and complex conditions (e.g., providing seated or roll-on scales, larger text on the tablet, etc.).

Further, as the technology has to be installed near an active phone line or internet connection and in an accessible space, choices were often limited in terms of equipment location. This meant that some patients had to keep it in a non-preferred location that is less accessible, in a common area where it can seem like an obstruction (e.g., kitchen), or a carpeted area on which the scale does not take accurate readings. Promisingly, many of the barriers to the technology use are being addressed over time and with troubleshooting, resulting in increased accessibility across the LHINs (e.g., with cellular installation or modifications to the scale for use on carpet).

### Patient-level: alignment between patient abilities and program involvement

The Telehomecare program aims to “inspire individuals to manage their health at home” [43]. An overarching theme at the patient-level was that the degree to which patients participated in the program commonly related to their motivation and ability to do so. Motivation was a central facilitator in a patient’s full involvement in the program and its related activities. On the other hand, language barriers significantly hindered patient participation.

Most stakeholders expressed how patient motivation was significant to a patient’s participation in and potential benefit from the program. The influence of and contexts shaping motivation were evident in both observations and interviews with patients. Generally, patient motivation or enthusiasm to participate was related to a view that the program could help address their major health concerns. Firstly, almost all of the patients and informal caregivers underscored the positive potential of the program, particularly with respect to advancing awareness and management of patient health as well as having immediate access to a health care provider when needed. This contributed to a sense of security for most patients and informal caregivers as they felt they could better manage their health at home, and reduce reliance on primary and emergency care for their health concerns.

*“When one was discharged from the hospital there never used to be real follow-up… You felt like you were being dropped off a cliff… [Telehomecare] provided a sense of security, because you were in contact with someone who could help you, versus having to make an appointment with your family physician, and get there, and get back…” – Patient 139*

This feeling of security was particularly noticeable among patients and informal caregivers who experienced difficulties in accessing a primary care provider when needed.

Conversely, many patients and informal caregivers who felt the program content was not very relevant to their needs seemed less motivated to participate. These patients were often already connected to health service supports for their conditions and regularly monitoring their health, or experiencing complex conditions that they felt could not be affected by the self-management guidance provided in the program. For example, one informal caregiver felt the advice given during the daily questionnaire was often not relevant to her husband who was not very mobile:

*“Skip your housework…don't go shopping…that kind of stuff, you know… I laugh at the questions because none of them pertain to him [laughs].” – Informal Caregiver to Patient 030*

Lastly, communication between patients and Telehomecare health care providers affected the majority of patients’ satisfaction with and motivation to continue the program. Many patients and informal caregivers were very satisfied with the Telehomecare nurses with whom they communicated, underscoring their appreciation of the information and support provided:

*“It was very comforting… Sometimes just doing the whole regime first thing in the morning got a little under my skin. But I did it anyways. And then by talking to them and just by realizing somebody else did care, it helped and I followed it through right to the very end.” – Patient 097*

This motivation to ‘follow through’ was often seen among patients and informal caregivers who said they were consistently communicating with and receiving encouragement from their designated Telehomecare nurse.

On the other hand, communication barriers related to language could hinder a patient’s capability to participate in the program. This was emphasized by stakeholders across all levels. Specifically, the Telehomecare equipment and literature have only two language options - English and French – yet there is a high level of ethnolinguistic diversity across all regions of study (e.g., Punjabi, Italian, Aboriginal languages). Many Telehomecare stakeholders said that, although interpretation services are technically available to them for enrollment and coaching sessions, it was not feasible or possible to use this service in all program-related interactions (e.g., during technician training, technical troubleshooting, etc.). Consequently, informal caregivers, particularly sons and daughters of those in the program, were often the point of contact for Telehomecare technicians and health care providers, as well as the operator of the technology. Given this reliance on informal caregivers, and the fact that only some nurses were multi-lingual, language barriers could result in a potential patient being ineligible for the program.

*“It’s a huge challenge, the language barriers. Some want to be in the program so badly but the language barrier is so bad. We do have interpreters but how feasible is that…how is the interpreter going to go into the home and look at questions and answer the questions, you know?… It's a hard decision to make…if we will accept them into the program because of that language barrier.” – Administrator 004*

Importantly, this barrier has been recognized by OTN and they are said to be strategizing on how to expand language options for patients.

### Provider-level: alignment between health care provider roles and capacities to fulfill them

For Telehomecare nurses and administrators, the program involves a high level of patient data management and monitoring, continuous communication with Telehomecare staff, technicians and patient primary care providers, and regularly providing care and coaching for patients. A central facilitator that allowed for many of these stakeholders to participate in the program to their fullest capacities was that they felt supported in their Telehomecare-related activities. In contrast, the largest barrier to a provider’s capacity to fulfill their role was related to time limitations.

Telehomecare nurses commonly highlighted that being supported to fulfill their roles was central to job performance and satisfaction. A key form of support included administrative assistance, as many felt that some of the activities were tangential to their role as nurse (e.g., troubleshooting technology with patients, paperwork, etc.). One nurse explained how her initial expectations for her role conflicted with the administrative tasks required of her:

*“When I was told what my role was, I was very excited in terms of doing a lot of work in health coaching, health teaching, just be with a patient for my 8 h shift, connect with them all the time… There were a lot of other responsibilities like a lot of paper work, and sometimes it takes so much of the time that you could spend with the patient and I was thinking, okay, I am supposed to really help the patients or do all these things which is not really nursing work.” – Telehomecare Nurse 003*

Though not integrated in all regions from the beginning of the program, designated Telehomecare team assistants who are responsible for administrative tasks are now considered central to Telehomecare nurse support and job satisfaction:

*“They are the tie that binds everything… they are identifying eligible patients…receiving calls from patients, and doing phone call support if they are having trouble with their unit… They are more of a program coordinator I would say, and extremely integral.” – Decision Maker 007*

Another form of support Telehomecare nurses underscored as important was feeling they could communicate with or receive help from co-workers, other health care providers, and managers when required. This included the ability to provide feedback on Telehomecare-related issues and engage in knowledge exchange with other Telehomecare stakeholders. For instance, job satisfaction seemed particularly high (and staff turnover low) in one LHIN, where timely support and opportunities to communicate with both management and other nurses on a scheduled and unscheduled basis, were evident. In another LHIN providing Telehomecare, a lack of communication regarding program-related challenges may have played a role in the high staff turnover, particularly at the beginning of the program’s implementation.

Closely linked to the challenges that can arise with a lack of support in Telehomecare health care provision is an insufficient amount of time to perform one’s expected duties. All Telehomecare nurses interviewed underlined how their patient caseloads were too high due to a set patient quota of 80 to 100 patients for all regions. Many expressed concern that this quota was a barrier to completing all expected tasks. This placed a great deal of stress on many nurses, who felt that their time limitations could hinder patient care. Specifically, they felt they did not have enough time to connect with and coach their patients while also completing a number of other required activities:

*“They say when you do health coaching your phone call should only last 7 min in order to maintain a caseload of 60 people… Well…it’s just not feasible when you’ve got some of the clientele we have.” – Telehomecare Nurse 064*

Notably, this issue prompted one LHIN to reduce their quota to 55 to 60 patients. This was influenced by a greater realization of a large discrepancy between expected time estimates set for each activity and actual time required:

*“[The original caseload] was based on the assumption that nurses would spend 5 % of their time on alert management [contacting patients whose data is outside of ‘normal’ range]… Well OTN is doing some analysis and we are finding that the nurses spend up to 25 % of their time on those alerts.” – Administrator 012*

Perhaps reflective of time limitations experienced by Telehomecare nurses, is the fact that none of those interviewed mentioned using the online communication portals made available for them by OTN. Further illustrating these limitations are a small number of patients’ remarks that they do not participate in weekly coaching sessions, and tend to only speak with their nurse when there is an urgent health alert.

### Organization-level: alignment between organization objectives and the current health system

The objectives of the Telehomecare program and what it involves were discussed by many stakeholders and found within the document review. A central theme at the organization level was a common disconnect between Telehomecare objectives (and related protocol) and current health care delivery. A key facilitator to meet Telehomecare objectives was related to improving integration of Telehomecare across health care providers and programs, while a major barrier was a lack of access to appropriate health care for certain patients.

Integrating the Telehomecare program into current models of primary care delivery was often seen by health care providers, decision makers, and administrators as essential to the success of the program. In general, when physicians were knowledgeable about the program, they were highly supportive and encouraged patients to become involved. However, a high number of physicians and specialists were not engaging with the program, particularly near its outset. Many Telehomecare nurses found that they often had little to no contact with their patient’s primary care provider, beyond sending progress reports to them. Further, for this study, the response request to physician interviews was extremely low. The likely reasons for this lack of involvement (cited by a number of stakeholders) were interrelated: a) not knowing about the program, or b) not having the time or incentives to become involved. As one decision-maker emphasized:

*“… [Primary care providers] are just busy… It feels to them like an add-on instead of an integrated approach… It is one more change, one more thing, and it is not integrated into their health records and into their health system.” – Decision Maker 014*

Over time, primary care provider awareness and involvement in the program has developed, particularly where integrated care models exist, such as Family Health Teams (FHTs). For instance, one decision maker describes how her rural FHT streamlined the Telehomecare referral and care process in ways that reduced the time restraints physicians might otherwise experience due to their involvement. This involved allowing other members of the FHT to refer potential patients and having RPNs (Registered Practical Nurses) funded by the host organization, assist with patient monitoring and communicate important patient updates to physicians.

“…*the more you can design a program, attaching it to a Family Health Team’s flow, you’re going to get a bigger buy-in and more of a clinic commitment than [the host organization] sending out the package and saying ‘we’re running this new service – start sending referrals.’” - Decision Maker 164*

Some physicians also emphasized the importance of provider collaboration for the purposes of integrating Telehomecare related processes right into their workflow:

*“If you want to build collaborative teams who actually talk to each other and have two way conversations, having people linked and embedded [in a Family Health Team] is better, way better… Our CCAC care coordinator will come right down to the clinic… It’s way simpler than before when we used to have to remember who to phone, then play telephone tag to try to reach them, and then explain what we need. I mean it’s a night and day difference.” – Physician 167*

Another decision maker described how the integration of Telehomecare into a Chronic Disease Management Framework in one LHIN has created more personalized health care supports for patients:

*“[Telehomecare] can’t be an island. It has to link to other things that we do to help to increase the impact… Maybe we can divert [patients] to a respirology clinic for rapid assessment and that keeps them out of ED, but really respond quickly….You also connect people to programs…and help them to prevent worsening of their conditions” – Decision Maker 011*

Importantly, the program’s potential to promote the integration patient care was often argued to be a necessary step to transform the health care system into one that is more effective, efficient, and patient-centred.

Despite the ability for the program to connect to a high volume of patients with COPD and HF, barriers existed in providing potential patients with appropriate care. This was in part related to varied interpretations of the ‘target’ population. For example, some stakeholders believed that the program should not target very complex or end-stage patients as they would not be able to self-manage during or after the program:

*“We do have some end-stage patients, but we try to shy away from that simply, because we know that we are not going to get a lot of success. And they will be not able to meet the parameters of the program.” - Telehomecare Nurse 025*

By this rationale, many potential patients who experience complex health conditions and do not have regular assistance in their daily activities may be overlooked. On the other hand, some stakeholders saw the program as an opportunity to connect potential patients to health care that they may not have otherwise. In this case, the program is considered to be more than a facilitator to self-management - it ensures timely access to health care more generally:

“*It’s not just self-management. It is self-management up to the point when you have difficulties self-managing. It’s also getting to a primary care provider hopefully earlier and faster than you did before…” – Decision Maker 023*

*“… now we are taking [patients waiting for long-term care] because we realize that some of them are waiting for a long time like 2 years, 3 years to go to nursing homes…” – Telehomecare Nurse 065*

The Telehomecare program thus becomes an important resource for patients and informal caregivers who may otherwise lack appropriate health care supports. For such reasons, many stakeholders expressed concern for the welfare of patients after they are discharged from the program:

*“We’re creating a program to divert them from emerge, we’re pulling the equipment out so they no longer can use it, and when things get rough, just go to emerge. Then why did we start this? We’re right back to where we started.” – Decision Maker 164*

*“A lot of these programs including ours, are for a period of time… the question is then, what happens to these patients?… We pray that they learned all they need to…but then what happens?” – Administrator 010*

This concern and the need to better address the potential gaps in patient care was a key theme across stakeholders.

### Structural-level: alignment between structural contexts and program operation

Though not always explicitly stated within interviews and observations, structural contexts significantly shape how the program is implemented and adopted. Governmental and policy-related support of comprehensive program evaluations was highlighted as a necessary facilitator to the effective implementation of Telehomecare. In contrast, macro-level factors such as geography and social location were potential barriers in connecting some patient populations to the program.

Governmental and policy contexts directly influence how the program has been evaluated, presenting both challenges and opportunities for Telehomecare. In line with government funding cycles, evaluative focus has predominantly been on meeting shorter-term outcomes and deliverables (e.g., reducing emergency department visits, reaching quotas) and ensuring this is cost-effective. Some stakeholders discussed the tension between government funding and evaluation frameworks, and conducting comprehensive program evaluations:

*“They do this kind of stuff too early in the project cycle, and they don’t give enough time for the thing to be fully implemented, fully evaluated, because government have a time frame that is unrealistic… we have hard measures and we miss the other ones, the softer ones. … It doesn’t give enough time to measure with patients, what difference has it made to you, and your knowledge of your disease. The number of ED visits does not change, but maybe because you measure it in 6 months instead of year.” – Decision Maker 014*

Promisingly, many stakeholders highlighted that local-level evaluations and observations reflect the positive potential of Telehomecare on patient health and emergency department visits. However some stakeholders, particularly decision makers, spoke of a need to move beyond individual factors such as patient enrollment or technology use, to fully evaluate whether and how this relatively new way of providing care is working for patients and providers. One decision maker emphasized that such evaluation has to occur to facilitate stakeholder buy-in and integrate Telehomecare on a large scale:

*“Telehomecare is not a technology…. It’s a care model that just happens to use technology. I think that’s where we get caught…. The government’s driving it, but they’re looking at it like a technology - ‘So, what have we saved?’ - so already we’re set up for failure.” – Decision Maker 013*

Overall many study participants felt that the impact of the Telehomecare program needed to be assessed with multiple stakeholders and in multiple locations. Notably, the majority of participants were very interested to learn the results of the mixed method programmatic evaluation of Telehomecare, which includes this qualitative study.

Other key structural-level factors that can potentially impact Telehomecare are related to geography and social location. Great distances to health care services and harsh climates in the largest LHIN of 400,000 km^2^, was often a barrier to health care access in general, and Telehomecare engagement specifically. Importantly, patients and informal caregivers on the program and living in areas where access to a provider was limited appreciated how Telehomecare made it possible to immediately access health support. According to other stakeholders, travel time and costs associated with Telehomecare nurse home visits (which occurred in the early stages of the program), technician installation, and technology trouble-shooting are very high in this LHIN:

*“It’s been extremely expensive, extremely awkward and…we end up having to pick the low hanging fruit and sort of concentrate on…those communities that are a little bit more densely populated but then we’re not getting the people who need the care the most.” – Decision Maker 007*

Stakeholders in this region in particular highlighted the need for program resources, timelines, and evaluations to take into account how geography and local-level contexts shape the implementation and uptake of the program.

Beyond geography, a number of social locations were mentioned or observed as potentially influencing patient perception, compliance and care. These included one’s age, culture, support networks, socioeconomic status, literacy level, ability, ethnicity, immigration status, and food availability. For instance, some health care providers said that First Nations may be hesitant to engage with the program, or may feel that medical models of care do not align with more holistic culturally-based approaches to health and wellness. In addition, socioeconomic status could prevent potential patients from adequately self-managing their conditions. For example, an inability to afford health-related resources (e.g., medications, monitoring equipment, transport to health care facilities, etc.) was seen to increase stress and hinder one’s ability to participate in self-management activities.

## Discussion

This multi-level analysis suggests that, despite the clear potential for Telehomecare to positively impact the lives of individuals affected by chronic conditions as well as strengthen the ways in which health care can be provided, a number of key challenges remain. Our findings largely reflect some of the key themes found in qualitative studies of similar programs, and in particular, that of the most recent large-scale programme evaluation of Telehomecare to date in the UK: the Whole Systems Demonstrator (WSD) Programme [16, 19, 21]. An overarching theme shared between this study and the WSD Programme study is the importance of aligning Telehomecare to the contexts and capacities of all stakeholders involved. This can facilitate increased buy-in and investment across stakeholders, and contribute to the success of the program.

Common facilitators shared by this and other studies include: patient motivation and ability to participate - particularly if they sought quicker access to health care [11, 44], user-friendly technology [45]; consistent communication between stakeholders [19], and collaborative and integrative care models [16, 19, 46]. Common barriers to Telehomecare largely derive from a disconnect between the overarching vision and goals for the program, and stakeholder needs. These barriers include language barriers [21], inadequate time and resources [20], a push to expand implementation before integrated stakeholder supports are in place [19], organizational and professional agendas [16, 46], short-term funding and evaluation cycles [19] and a limited evidence base – particularly in terms of local evaluations [19, 20].

A major conclusion deriving from the study results is that Telehomecare can help strengthen and be strengthened by integrated health care models. For instance, the program worked well when integrated with collaborative models and programs, such as Family Health Teams (community-centred primary care organizations with tailored services for the populations they serve) and MOHLTC’s community Health Links (a program working to improve the coordination of care to patients with complex needs). Notably, these practices can foster consistent stakeholder communication and input into health care processes. Such models have increasingly shown their potential to: a) reduce gaps in patient health care (e.g., linking patients to health care providers and services), particularly for populations who face disproportionate barriers to health care; b) reduce duplication in patient health care provision; and c) facilitate health care communication and support for multiple stakeholders [47–50]. In this, integrated care models can help reduce some of the multi-level barriers to Telehomecare implementation and adoption seen in this study and beyond, including a lack of stakeholder buy-in and capacity, and, at the patient level specifically, barriers to health care access. Addressing such barriers via improved care integration was recently highlighted by Health Quality Ontario as essential to ensure diverse populations get the care they need [51].

In working towards improving coordinated patient care, it is vital that further implementation and expansion of the Telehomecare program involve regular needs assessments as to what is currently working and not working across all stakeholders. This must take into account the ways in which Telehomecare processes and goals converge or contrast with the day to day abilities and goals of stakeholders. As Hendy et al. [19] argue, programs like Telehomecare need to be driven by the needs of stakeholders to ensure they have a meaningful impact from the individual level to the societal level. As such, continued dialogue and knowledge exchange among and between stakeholders surrounding how to best integrate the Telehomecare program across local and regional contexts needs to occur. This is reflected in the recent call to implement a “‘top-down’ mandate to innovate from the ground-up” [52] in the process of integrating care for people with chronic health and social needs in Canada. Importantly, innovating Telehomecare from the ground up would allow for increased flexibility in care processes that can adapt to local contexts and capacities. Doing this requires a commitment to local level evaluations that include the perspectives of community stakeholders across multiple levels. This can allow for stronger links to be made across levels; for example, between patient health, health behaviour and social contexts. As this study demonstrates, evaluations across micro-, meso- and macro-levels can facilitate a comprehensive view of how Telehomecare is working, and thus a comprehensive response to address what is not working.

At a more fundamental level, the study findings reflect a need to discuss and be transparent about the goals and objectives of Telehomecare. As a Telehomecare pioneer recently emphasized, limited and varied conceptualizations of what Telehomecare is and does has also limited its implementation [53]. Specifically, he argues that Telehomecare has been framed as an individual-level technology, rather than a tool to support organizational or systemic change. Because of this, the impetus to plan, implement and evaluate Telehomecare as centered on care integration has not been significant, and correspondingly, effectiveness and buy-in across stakeholders is low [25]. In light of this, reframing Telehomecare as an innovative model of care requiring the input and involvement of multiple stakeholders could help bridge the gaps between the Telehomecare program, the health care system, and the broader contexts shaping health, and above all, improve population health care and well-being.

### Strengths and limitations

This study expands on current knowledge as to the barriers and facilitators to Telehomecare implementation and adoption. Its strengths lie in the utilization of triangulated data collection and analysis that allowed for an in-depth and context-rich synthesis of themes across five levels of Telehomecare. Notably, the analysis integrates structural-level considerations, such as socioeconomic contexts, which are relatively absent from the current evidence base. Macro-analysis of this sort is particularly important to understand the multidimensional contexts in which health care and health care systems are situated, as well as identify the interrelated factors influencing individual health that may not otherwise be considered within health care research and practice [54, 55]. In addition, the results of this qualitative study will be triangulated with the two quantitative components of the program evaluation, allowing for evidence on effectiveness and cost-effectiveness to be comparatively evaluated with and informed by the qualitative findings, and vice versa. Such mixed-method and multi-level approaches have been argued as vital to ensure health care research produces “real-world” findings that are relevant across stakeholders and settings [55].

It is important to underscore that our study participants were a sample of Telehomecare stakeholders and thus did not represent all stakeholder views and contexts. This is not recognized as a significant limitation, as the goal of qualitative inquiry is not to seek representativeness in its findings, but rather to contextualize findings. In addition, as the scope of this paper only allowed for the overarching findings across the regions of study to be highlighted, it is important that further details (including a comparative analysis across the LHINs) be reviewed by interested stakeholders within the original Qualitative Comparative Case Study Report [56]. Lastly, given the preliminary nature of the program, it is imperative that continuous effort is made to better understand and address the needs of diverse Telehomecare stakeholders across regions and social locations. This includes seeking the input of stakeholders who are not engaged, or have declined participation in the program to illuminate potential barriers to involvement that are being overlooked. This can ensure that program planning, implementation, and expansion can best reflect the needs and capabilities of all people who are involved or could be involved.

## Conclusion

This qualitative inquiry makes an important contribution to understandings of potential facilitators and barriers to Telehomecare. Consistent with studies of similar programs elsewhere, the success of this Telehomecare program is dependent on a range of multi-level factors across multiple stakeholders. As illustrated by the study findings, consistent evaluation and dialogue surrounding what works and what does not work is central to ensuring the program is accessible, effective, and sustainable. In particular, these processes must prioritize the input of both potential and current stakeholders. With continued qualitative and mixed-method evaluation and sharing of best practices, the Telehomecare program and others like it can evolve and expand in ways that align with the needs and goals of all who participate.

## Additional file

Please see “Additional file 1” titled “Semi-structured interview guides.” This includes the interview guides used for all stakeholder interviews (patients, health care providers, administrators, decision makers and technicians). Additional file 1:
**Semi-structured interview guides.** (PDF 409 kb)

## Abbreviations

CCAC: Community Care Access Centre
FHT: Family Health Team
LHIN: Local Health Integration Network
MOHLTC: Ontario Ministry of Health and Long Term Care
OTN: Ontario Telemedicine Network
