# Supplementary material for: A multi-level qualitative analysis of Telehomecare in Ontario: challenges and opportunities
Source: BMC Health Serv Res. 2015 Dec 9;15:544. doi: 10.1186/s12913-015-1196-2 (PMC4673764; doi:10.1186/s12913-015-1196-2)
Supplement: Additional file 1: — Semi-structured interview guides. (PDF 409 kb) [file 12913_2015_1196_MOESM1_ESM.pdf]

Semi-structured interview guides

Patient Interview Guide

Patient Enrolment / Patient Care Delivery

1. Opening Question: Can you tell me what your health condition was like before enrolling in THC?
    a. What did you do to manage your care?
    b. Who did you turn to for help/advice?
    c. What are your particular health concerns?

2. What are your expectations and views on purpose of the THC program/service
    a. Based on what you have been told, what are your expectations of the THC program/service?
    b. Did any expectations and views change since enrolment debriefing?
    c. What aspects of the program are you looking forward to? Not looking forward to?

3. Can you tell us more about your experience with the initial installation of THC in your home?
    a. Did you find it helpful and do you feel you have access to the support you may need while using the monitoring device throughout your participation?

4. Were you aware of services such as the THC program/service before being approached?

5. Exploration of what happened during the THC program/service review (using observation notes).

6. Do you have any concerns about your medications while being part of the THC?

7. Has your relationship changed in any way with your GP and other members of care?
    a. Concerns about GP and other health professional role in patient's care?
    b. Concerns about GP and other health professional role with patient caregiver?

8. Program Adherence
    a. Do you find the THC model is helpful to increase your self-management skills? If yes, can you give us a specific example?
    b. What specific characteristics help make you more responsive in terms of self-management skills?

Patient/Caregiver Overall Views

1. Some of the most enjoyable or least enjoyable parts of THC (referral, technology delivery, initial training, maintenance, care transitions, etc.).

2. Improving the service / another THC program/service in future?

3. What were your feelings regarding the THC technology (i.e. past, current, future?)

4. If you are the informal caregiver to the patient, do you perceive the functionality of the technology to support aspects of your family member's immediate health care needs (e.g. changes in medications, activity/lifestyle practices, health care access)?

5. What other types of support have you received since your family member has been enrolled in this project?

6. What unexpected barriers (e.g. language) exist that prevent the patient from making productive use of equipment or having productive interactions with the THC nurses?

7. What technological limitations (hardware or software) appear to inhibit the productive use of equipment by patients?

8. Enjoyable or least enjoyable parts of THC (technology delivery, training, maintenance, care transitions, discharge, etc.).

9. Please rate how you find the frequency of counselling appointments or meetings with the THC nurse from 1 to 10 (too often or not often enough)

10. How do you feel about discharge/any changes you would suggest?
    What will happen next? What were you told?

11. Is there anything else that you would like to comment on about the THC model that you feel is relevant for us to know?

Health care Provider Interview Guide

1. Opening Question: Could you tell me from beginning to the end your experience of the THC program/service in as much detail as possible?

2. Previous Health care provider experience
    a. Have you previously delivered primary care before joining THC?
    b. If yes, for how long?
    c. If yes, what setting have you mostly worked in?
    d. How would you describe your usual method of care delivery?
    e. How does your usual method of care delivery from previous experiences differ with your current approach to care delivery, remote health coaching and patient education?

3. Role within THC model
    a. Can you describe what you would do on a typical day?
    b. How does this differ from what you were told in the beginning about your role within THC?
    c. What changes would you make to your role?
    d. How did you learn to use Telehomecare technology?
    e. Were you involved in the decision to develop or implement any part of the THC model?
    f. Can you tell me about how care for patients is coordinated amongst the THC team?
    g. If you are a Primary Care Provider (PCP), what changes would you make to the way you connect with other members of the THC team and/or members of the patients' circle of care?
    h. If you are a member of the THC team, what changes would you make to the way you connect with other members of the THC Team and/or other members of the patients' circle of care?
    i. How do THC nurses encourage remote health coaching and patient education? Do any strategies (either organizational or individual) appear to be particularly effective?
    j. What is the process leading to a patient being enrolled in this project?
    k. What does a typical admission and follow-up look like in this care model?

4. Patient expectation of THC
    a. Does THC affect how patients understand their condition? Why do you say that?
    b. Do you know what your patients were told about the THC model? What challenges (if any) have you faced in your patients' expectations compared with the care you deliver?
    c. What characteristics of, or adaptations made by Telehomecare patients and/or caregivers appear to make them more responsible to self-management and as a result most likely to be successful in the program?

d. Based on your observation when you connect with patients, what aspects of THC do they enjoy?
e. Considering the patient experience end-to-end, what are some of the most and least enjoyable parts of THC (referral, technology delivery, initial training, maintenance, care transitions, discharge etc.)?

5. Interaction with patients
    a. What unexpected barriers (e.g. language) exist that prevent patients from making productive use of the equipment or having productive interactions with the THC nurses?
    b. In general, what has your experience been like of providing care for individuals with complex conditions? What are the challenges specific for this population?
    c. What tools do you use to help your patients better understand their condition? (e.g. pamphlets, brochures, etc.) What's been your experience of working with these new tools?
    d. Has your relationship with the client or their family members changed through the use of this technology and care model?
    e. Based on your observation, what are some of the challenges patients face? How can the THC model improve upon these challenges?

6. Other
    a. What technological limitations (hardware or software) appear to inhibit the productive use of equipment by patients?
    b. Is there anything else that you would like to comment on about the THC model that you feel is relevant for us to know?

Administrator Interview Guide

1. Opening Question: How did you become involved in this project?

2. Previous Administrative experience
    a. What was your previous experience with Telehomecare technology?

3. Role within THC model
    l. What is the typical process leading to a patient being enrolled in this project?
    m. What are your responsibilities/roles in the care delivery process?
        i. How does this differ from what you were told in the beginning about your role within THC?
        ii. What changes would you make to your role?
    n. What are the largest/smallest impacts to your role as a Health care Program Manager from the use of a Telehomecare care model?
    o. What training or other preparation would you have wanted to better prepare for your role?
    p. Were you involved in the decision to develop or implement any part of the THC model?
    q. How do you select a patient for admission into this project?
    r. What does a typical admission and follow-up look like in this care model?
    s. Can you tell me about how care for patients is coordinated amongst the THC team?
    t. Which other members of the THC team do you connect with? How and for what reason?
    u. Has your relationship with staff, clients, and their family members changed through the use of this technology and care model?
    v. What changes would you make to the way you connect with other members of the THC team?
    w. What types of resources and requirements are utilized (that are different) than your previous care delivery models?

    2. Patient expectation of THC
        a. Does THC affect how patients understand their condition? Why do you say that?
        b. Do you know what patients were told about the THC model?

    3. Administration for THC
        a. Which elements played a key role for successful implementation and adoption of THC?
        b. What mechanisms are in place for continuous improvement of THC?
        c. How do leaders implement THC and create a sense of business ownership, accountability, shared roles and responsibilities?

4. Other
    a. How do key elements of the Telehomecare model interact with other members of the patients' care teams (e.g. the effect on patient relationship with the primary care providers (PCP) or THC nurse interactions with PCP?)
    b. How are Telehomecare nurses trained and equipped to perform their duties? Are there any strategies (either organizational or individual) to promote effective intra-team coordination and interaction with senior managers? What additional training is required by the THC nurses to ensure that they are being prepared to provide fulsome service?
    c. How do THC nurses encourage remote health coaching and patient education? Do any strategies (either organizational or individual) appear to be particularly effective?
    d. Do any processes for delivery, storage and configuration of equipment to patients (either organizational or individual) appear to be more or less effective than others?
    e. In your opinion, what are the pros/cons of this technology and care delivery model?
    f. Is there anything else that you would like to comment on about the THC model that you feel is relevant for us to know?

Decision Maker Interview Guide

1. Opening Question: What are your responsibilities/roles in the management of care delivery processes?

2. Have these responsibilities changed/evolved with the addition of Telehomecare technology?

3. What does a typical admission and follow-up look like in this care delivery model?

4. What types of resources and requirements are utilized (that are different) than your previous care delivery models?

5. Which elements of a LHIN's or host organization's comprehensive implementation and adoption plan appear to most successfully encourage implementation and adoption of Telehomecare?

6. Have any LHINs or host organizations developed mechanisms for continuous improvement of some aspect of Telehomecare (e.g. lessons learned documents, staff focus groups, etc.)? Which ones appear to be the most effective? How have adaptations been made in response to these learning mechanisms?

7. How do LHINs and host organizations reach out to primary care providers and other referrers? Which strategies appear to be effective? What are the barriers to primary care providers and other referrers that are referring their patients to Telehomecare?

8. How do leaders of LHINs and host organizations champion the system-wide change required to fully implement Telehomecare? Do some leaders communicate this more successfully and, if so, what do they do? Have any LHINs developed strategies to tie Telehomecare to other priorities (e.g. Seniors Strategy, ALC reduction, etc.)?

9. How do LHINs create a clear sense of business ownership, accountability and shared roles and responsibilities for Telehomecare? Which strategies for this appear to be most effective?

10. In your opinion, what are the pros/cons of this technology and care delivery model?

Technician Interview Guide

1. Opening Question: Can you tell me from beginning to the end your experience of the THC program/service in as much detail as possible?

2. Previous Technician experience
    a. Have you previously provided technician/installation support for patients before joining THC?
    b. If yes, for how long?
    c. If yes, what setting did you work in?

3. Role within THC model
    a. Can you describe what you would do on a typical day?
    b. How does this differ from what you were told in the beginning about your role within THC?
    c. What changes would you make to your role?
    d. Were you involved in the decision to develop or implement any part of the THC model?
    e. Which other members of the THC team do you connect with? How and for what reason?
    f. What changes would you make to the way you connect with other members of the THC team?

4. Patient expectation of THC
    a. Do you know what your patients were told about using the THC model? What challenges (if any) have you faced in your patients' expectations compared with the information you deliver?

5. Interaction with patients
    a. What technological limitations (hardware or software) appear to inhibit the productive use of equipment by patients?
    b. Based on your observation when you connect with patients, what aspects of THC do they enjoy?
    c. Based on your observation, what are some of the challenges patients face? How can the THC model improve upon these challenges?

6. Other
    a. Is there anything else that you would like to comment on about the THC that you feel is relevant for us to know?
